# Comprehensive exposure assessments from the viewpoint of health in a unique high natural background radiation area, Mamuju, Indonesia

**DOI:** 10.1038/s41598-021-93983-2

**Published:** 2021-07-16

**Authors:** Eka Djatnika Nugraha, Masahiro Hosoda, Kusdiana, Untara, June Mellawati, Nurokhim, Yuki Tamakuma, Abarrul Ikram, Mukh Syaifudin, Ryohei Yamada, Naofumi Akata, Michiya Sasaki, Masahide Furukawa, Shinji Yoshinaga, Masaru Yamaguchi, Tomisato Miura, Ikuo Kashiwakura, Shinji Tokonami

**Affiliations:** 1 Center for Technology of Radiation Safety and Metrology, National Nuclear Energy Agency, Jl Lebak Bulus Raya No 49, Jakarta Selatan, DKI Jakarta 12440 Indonesia; 2 Department of Radiation Science, Hirosaki University Graduate School of Health Sciences, 66-1 Hon-cho, Hirosaki, Aomori 036-8564 Japan; 3 Institute of Radiation Emergency Medicine, Hirosaki University, 66-1 Hon-cho, Hirosaki, Aomori 036-8564 Japan; 4 Nuclear Fuel Cycle Engineering Laboratories, Japan Atomic Energy Agency, 4-33, Muramatsu, Tokai-mura, Naka-gun, Ibaraki, 319-1194 Japan; 5 Japan Nuclear Technology Research Laboratory, Central Research Institute of Electric Power Industry, 2-11-1, Iwadokita, Komae, Tokyo 201-8511 Japan; 6 Graduate School of Engineering and Science, University of the Ryukyus, 1 Senbaru, Nishihara-cho, Okinawa, 903-0213 Japan; 7 Research Institute for Radiation Biology and Medicine, Hiroshima University, 1-2-3, Kasumi, Minami-ku, Hiroshima, 734-8553 Japan

**Keywords:** Environmental sciences, Natural hazards, Nuclear physics

## Abstract

Mamuju is one of the regions in Indonesia which retains natural conditions but has relatively high exposure to natural radiation. The goals of the present study were to characterize exposure of the entire Mamuju region as a high natural background radiation area (HNBRA) and to assess the existing exposure as a means for radiation protection of the public and the environment. A cross-sectional study method was used with cluster sampling areas by measuring all parameters that contribute to external and internal radiation exposures. It was determined that Mamuju was a unique HNBRA with the annual effective dose between 17 and 115 mSv, with an average of 32 mSv. The lifetime cumulative dose calculation suggested that Mamuju residents could receive as much as 2.2 Sv on average which is much higher than the average dose of atomic bomb survivors for which risks of cancer and non-cancer diseases are demonstrated. The study results are new scientific data allowing better understanding of health effects related to chronic low-dose-rate radiation exposure and they can be used as the main input in a future epidemiology study.

## Introduction

Daily human life routines cannot be separated from radiation exposure, especially natural radiation exposure. The radioactive elements in the earth's crust that lead to human exposure are potassium (^40^K), uranium (^238^U), thorium (^232^Th), and their radioactive decay products, e.g., radium (^226^Ra) and radon (^222^Rn)^1–3^. Residents in some areas are exposed to radiation doses that are higher than the global annual mean radiation dose (around 2.4 mSv) by one to two orders, and a few regions are high natural background radiation areas (HNBRAs) with potential annual effective doses which are even higher than the 20 mSv dose limit for radiation workers^4,5^.

UNSCEAR reported^4^ that measurements in HNBRAs were important for radiation protection of their residents and for environmental radiation protection. Health studies of populations living in HNBRAs are a potential source of information on the effects of chronic low-dose-rate exposures. Although the studies of atomic bomb survivors provide strong evidence of health effects such as cancer and non-cancer diseases associated with single acute exposure to moderate to high doses of ionizing radiation, the effect of low dose-rates on health and cancer risks after exposure to ionizing radiation is, as yet, unclear. Based on these circumstances, it is necessary to make a comprehensive environmental assessment of the existing exposure situation in a HNBRA to get scientific evidence of health effects due to chronic low-dose-rate radiation exposure.

Some studies on HNBRAs of Brazil, China, India, and Iran have been made in recent years^4,6–9^. High radiation exposure levels can cause radiation-induced diseases such as cancer^10^. Additionally, it is important to note that the incidence of non-cancer diseases such as cerebrovascular diseases, tuberculosis, and digestive system diseases that was found in Yangjiang, China had a significant impact from the HNBRA^3,11^. As well as radiation exposure doses due to natural radionuclides, such contaminants as toxic heavy metals are important. Heavy metal pollution has been discussed in areas with high natural radionuclides in the soil, such as areas around uranium mines and HNBRAs^12^.

In 2015, the United Nations established the Sustainability Development Goals (SDGs) program for the sustainable development of human benefits including good health and well-being, clean water and proper sanitation as well as responsible consumption and production^13^. Natural radiation and heavy metals in the environment can be viewed as natural pollution. They can be present in the human body from both internal and external sources, and they can affect human DNA. In addition, natural radiation and heavy metals also enter the ecosystem (water, air, soil, plants and animals) naturally and by human activities so that the quality of life and the health of residents in HNBRAs are affected. This becomes a challenge for meeting the SDGs.

According to the ambient dose equivalent rate map of Indonesia, as shown in Supplementary Figure S1^14^, the ambient dose equivalent rate in Mamuju is quite high when compared to other regions. For example Takandeang Village had measured rates between 100 and 2800 nSv h^−1^^15^. This paper presents the first study that comprehensively explains exposure assessments for the whole Mamuju region from the viewpoints of health and human activities. We measured all parameters that contribute to external and internal radiation exposures caused by radionuclides, especially heavy metal radionuclides and including characterization exposure. Our goals were (1) characterizing exposure of the whole Mamuju region as for a HNBRA; (2) assessing the existing exposure to assure radiation protection of the public; and (3) providing the main input for an epidemiological study. This study is unlike previous studies that looked at several specific areas and had limited scope such as: the preliminary survey on Botteng Village, Mamuju^16^; the preliminary study on cytogenetics biomarkers^17^; radon (^222^Rn) and thoron (^220^Rn) measurements in Takandeang Village^18^; radioactivity measurements in soil samples and ambient dose equivalent rate in Northern Botteng Village^19,20^; radium measurements in drinking water^21^; and radioactive mineral explorations in Mamuju^22,23^. The limitation of this paper is that the dose contribution of ^210^Po has not been determined.

## Results

### Lifestyle and consumption behaviors

Generally, daily life in Mamuju is dependent on nature. Most residents work outdoors and consume food that is mainly locally grown or produced or that comes from traditional markets. The drinking water sources for almost 86% of the residents are wells and springs, and just a small percentage buy bottled drinking water. The drinking water habit is consuming it directly without boiling. Hence, the Mamuju residents are vulnerable to becoming contaminated by natural radionuclides and heavy metals, so it is important to measure the quality of drinking water, following the SDGs mandates to ensure cleanliness of drinking water and feasibility of sanitation. From our questionnaire, we found that the average consumption of drinking water per day was 1.6 L.

### Natural radionuclide activity concentration in soil samples

The concentration of natural radionuclides in soil samples in the Mamuju region is very high. The average activity concentrations of ^238^U, ^232^Th and ^40^K were 1387 Bq kg^−1^, 1468 Bq kg^−1^, and 301 Bq kg^−1^, respectively. The ^238^U activity concentration ranged from 570 to 3456 Bq kg^−1^, ^232^Th activity concentration ranged from 819 to 3577 Bq kg^−1^ and ^40^K activity concentration ranged from 121 to 555 Bq kg^−1^. Detailed information about the natural radionuclide activity concentration in soil samples can be found in Supplementary Figure S2 and Table S1.

### Drinking water quality and heavy metal element content

In general, drinking water quality in Mamuju is still below the reference level set by the Ministry of Health of Indonesia or the recommendation by WHO^24,25^. From the 30 samples we measured (Table 1), one sample had an electroconductivity above 1.5 mS cm^−1^. Drinking water samples had a pH range of 6.6–7.4; and electroconductivity ranged from 0.04 to 0.83 mS cm^−1^. For total hardness, chloride, sodium, sulfate, fluor and nitrate concentrations had ranges from 15 to 244 mg L^−1^, 0.02 to 6.87 mg L^−1^, 2.07 to 26.94 mg L^−1^, 0.08 to 8.46 mg L^−1^, 0.02 to 1.12 mg L^−1^, and 0.01 to 1.52 mg L^−1^, respectively. Most natural drinking water samples in Mamuju contained higher heavy metal concentrations than bottled drinking water did (Table 2).

**Table 1:** Drinking water quality measurement results of samples.

Sample number	Water type	Latitude (south)	Longitude (east)	Altitude (m)	Temperature (^o^C)	pH	Electric conductivity at 25 °C (mS cm^−^1)	Radon activity concentration		Total hardness (mg L^−1^)	Chloride (mg L^−1^)	Sodium (mg L^−1^)	Sulfate (mg L^−1^)	Fluoride (mg L^−1^)	Nitrate (mg L^−1^)
								Dry season (Bq L^−1^)	Rainy season (Bq L^−1^)						
1	Tap	2.77413	118.89231	776	31	7.4	0.92	617	31	32	0.55	10.79	0.68	0.22	0.01
2	Well	2.78188	118.86263	768	28	7.3	0.83	194	16	32	0.76	8.58	0.21	0.2	0.08
3	Well	2.77890	118.86933	775	27	7.0	0.16	100	18	102	0.96	22.67	8.06	1.12	0.05
4	Well	2.77168	118.86718	680	26	7.4	0.29	152	13	102	0.96	22.67	8.06	1.04	0.01
5	Tap	2.77789	118.86439	668	27	7.4	0.30	344	18	44	2.26	7.91	5.65	0.13	0.45
6	Well	2.77789	118.86447	671	26	7.3	0.30	333	18	68	5.18	7.85	6.84	0.09	1.52
7	Well	2.77811	118.86464	660	27	7.0	0.27	712	34	48	4.28	5.53	0.53	0.08	0.19
8	Well	2.77811	118.86351	750	27	6.6	0.25	1057	39	106	0.52	23.18	8.46	1.08	0.04
9	Well	2.77961	118.86400	645	27	6.4	1.25	1142	44	28	6.87	11.2	5.91	0.1	4.48
10	Spring	2.80878	118.88650	817	27	6.2	1.81	145	11	24	0.52	4.63	0.18	0.13	0.08
11	Well	2.80750	118.86761	737	26	7.1	0.04	39	6	25	0.87	7.16	0.34	0.12	0.19
12	Well	2.80570	118.86220	690	26	7.2	0.61	134	11	221	1.99	3.31	2.9	0.23	0.43
13	Tap	2.77433	118.81215	961	26	6.9	0.30	38	3	30	1.89	3.35	0.5	0.34	0.68
14	Tap	2.77450	118.81696	893	26	7.2	0.31	57	5	15	0.99	2.07	0.4	0.1	1.91
15	Tap	2.75886	118.85141	834	26	7.2	0.32	444	20	117	0.57	26.94	3.32	1.04	0.01
16	Well	2.75058	118.85583	760	27	7.1	0.29	16	2	244	0.7	2.28	2.32	0.17	1.65
17	Well	2.75020	118.85508	776	26	7.4	0.33	23	3	61	0.64	6.27	9.3	0.59	0.01
18	Well	2.75312	118.85666	751	26	7.3	0.33	117	9	241	0.93	2.41	2.25	0.16	1.71
19	Well	2.84733	118.81232	551	26	7.2	0.31	154	8	241	0.93	2.41	2.25	0.16	1.71
20	Well	2.84818	118.81026	543	26	7.1	0.35	143	8	241	0.93	2.41	2.25	0.16	1.71
21	Well	2.85315	118.87070	10	26	7.2	0.31	56	3	58	5.13	7.21	4.91	0.02	0.22
22	Well	2.85572	118.86898	15	26	7.4	0.32	66	4	56	4.6	7.24	4.91	0.02	0.22
23	Well	2.17331	119.22960	925	26	7.2	0.33	4	0.8	25	0.86	7.06	0.33	0.12	0.19
24	Well	2.17717	119.22865	826	26	7.3	0.29	4	0.8	28	0.97	7.94	0.38	0.13	0.21
25	Well	2.80378	118.86147	24	26	6.7	0.33	29	1	50	2.35	3.21	3.25	0.03	0.35
26	Well	2.79612	118.86265	25	26	6.8	0.32	35	2	45	2.12	3.25	3.95	0.02	0.31
27	Well	1.93528	119.36628	5	31	6.7	0.30	3	0.2	58	3.4	5.27	4.91	0.02	0.22
28	Well	1.94642	119.36285	5	31	6.7	0.30	2	0.2	58	3.4	5.27	4.91	0.02	0.22
29	Bottled water	-	-	–	26	7.1	0.16	1	0.2	25	0.02	3.63	0.08	0.13	0.06
30	Bottled water	-	-	–	26	7.0	0.16	1	0.2	23	0.02	2.63	0.1	0.13	0.05
Reference level					T* ± 3	6.5–8.5	1.56	11	11	–	250	200	250	1.5	50

T* = ambient temperature.

**Table 2:** Heavy metal measurement results of drinking water samples given in Table 1.

Sample number	Cr (ng L^−1^)	Mn (ng L^−1^)	Co (ng L^−1^)	Ni (ng L^−1^)	Cu (ng L^−1^)	As (ng L^−1^)	Se (ng L^−1^)	Cd (ng L^−1^)	Ba (ng L^−1^)	^206^Pb (ng L^−1^)	^207^Pb (ng L^−1^)	^208^Pb (ng L^−1^)	Total Pb (ng L^−1^)
1	(1.46 ± 0.03) × 10^3^	(4.14 ± 0.08) × 10^3^	< MDC	(2.62 ± 0.05) × 10^2^	(2.073 ± 0.41) × 10^3^	(1.11 ± 0.02) × 10^3^	(7.40 ± 0.10) × 10^1^	< MDC	(4.88 ± 0.98) × 10^5^	(5.48 ± 0.11) × 10^2^	(5.56 ± 0.11) × 10^2^	(5.55 ± 0.11) × 10^2^	(1.66 ± 0.03) × 10^3^
2	(1.91 ± 0.04) × 10^3^	(9.06 ± 0.18) × 10^2^	(3.46 ± 0.07) × 10^2^	(2.42 ± 0.05) × 10^2^	(2.04 ± 0.41) × 10^3^	(1.77 ± 0.04) × 10^3^	(3.7 ± 0.1) × 10^1^	< MDC	(5.21 ± 0.10) × 10^5^	(4.85 ± 0.10) × 10^2^	(4.90 ± 0.10) × 10^2^	(4.87 ± 0.10) × 10^2^	(1.46 ± 0.03) × 10^3^
3	(1.1 ± 0.02) × 10^3^	(1.08 ± 0.03) × 10^3^	< MDC	(6.26 ± 0.12) × 10^3^	(8.40 ± 0.17) × 10^4^	< MDC	(3.4 ± 0.1) × 10^1^	< MDC	(2.83 ± 0.06) × 10^4^	(3.49 ± 0.08) × 10^3^	(3.64 ± 0.07) × 10^2^	(3.57 ± 0.07) × 10^2^	(1.07 ± 0.02) × 10^4^
4	< MDC	(7.26 ± 0.15) × 10^4^	(3.11 ± 0.06) × 10^2^	(1.13 ± 0.02) × 10^2^	(1.26 ± 0.03) × 10^3^	< MDC	< MDC	< MDC	(4.64 ± 0.09) × 10^4^	(2.48 ± 0.05) × 10^2^	(2.62 ± 0.05) × 10^2^	(2.56 ± 0.05) × 10^2^	(7.66 ± 0.15) × 10^2^
5	< MDC	(1.97 ± 0.39) × 10^4^	(4.61 ± 0.09) × 10^2^	(9.0 ± 0.2) × 10^1^	(1.905 ± 38) × 10^2^	< MDC	(1.12 ± 0.02) × 10^2^	< MDC	(7.75 ± 0.16) × 10^5^	(1.76 ± 0.04) × 10^2^	(1.75 ± 0.04) × 10^2^	< MDC	(3.51 ± 0.12) × 10^2^
6	(1.78 ± 0.04) × 10^2^	(2.49 ± 0.50) × 10^4^	< MDC	(8.4 ± 0.2) × 10^1^	(5.96 ± 0.12) × 10^2^	< MDC	(1.07 ± 0.02) × 10^2^	< MDC	(3.91 ± 0.08) × 10^5^	< MDC	(8.70 ± 0.2) × 10^1^	< MDC	(8.70 ± 0.2) × 10^1^
7	(4.43 ± 0.09) × 10^2^	(4.63 ± 0.93) × 10^5^	(2.94 ± 0.06) × 10^3^	(5.99 ± 0.12) × 10^2^	(1.51 ± 0.30) × 10^3^	< MDC	(6.4 ± 0.1) × 10^1^	< MDC	(7.44 ± 0.15) × 10^4^	(1.25 ± 0.03) × 10^2^	(1.32 ± 0.03) × 10^2^	< MDC	(2.58 ± 0.06) × 10^2^
8	< MDC	(6.53 ± 0.13) × 10^4^	(1.62 ± 0.03) × 10^2^	< MDC	(9.17 ± 0.18) × 10^2^	< MDC	< MDC	< MDC	(5.26 ± 0.11) × 10^4^	< MDC	< MDC	< MDC	< MDC
9	(6.01 ± 0.12) × 10^2^	(3.91 ± 0.78) × 10^4^	(5.73 ± 0.11) × 10^2^	(5.33 ± 0.11) × 10^2^	(3.57 ± 0.07) × 10^3^	(1.59 ± 0.03) × 10^4^	9 ± 0.2	< MDC	(1.33 ± 0.03) × 10^6^	(1.15 ± 0.02) × 10^3^	(1.16 ± 0.02) × 10^3^	(1.15 ± 0.02) × 10^3^	(3.46 ± 0.07) × 10^3^
10	(1.96 ± 0.04) × 10^3^	(1,93 ± 0.04) × 10^3^	< MDC	(9.70 ± 0.2) × 10^1^	(2.42 ± 0.05) × 10^3^	(7.09 ± 0.14) × 10^3^	< MDC	< MDC	(1.58 ± 0.03) × 10^5^	(3.86 ± 0.08) × 10^2^	(3.89 ± 0.08) × 10^2^	< MDC	(7.76 ± 0.16) × 10^2^
11	(6.88 ± 0.14) × 10^2^	(1.72 ± 0.03) × 10^3^	(2.31 ± 0.05) × 10^2^	(1.47 ± 0.03) × 10^2^	(7.29 ± 0.15) × 10^2^	< MDC	< MDC	< MDC	(7.42 ± 0.02) × 10^5^	(3.92 ± 0.08) × 10^2^	(3.87 ± 0.08) × 10^2^	< MDC	(7.79 ± 0.16) × 10^2^
12	(4.41 ± 0.09) × 10^2^	(7.06 ± 0.14) × 10^4^	(2.10 ± 0.04) × 10^3^	(4.88 ± 0.10) × 10^2^	(3.44 ± 0.07) × 10^3^	(6.34 ± 0.13) × 10^3^	(1.11 ± 0.02) × 10^2^	< MDC	(1.19 ± 0.02) × 10^6^	(1.14 ± 0.02) × 10^3^	(1.18 ± 0.03) × 10^3^	(1.15 ± 0.02) × 10^3^	(3.47 ± 0.07) × 10^3^
13	(2.78 ± 0.06) × 10^2^	(4.01 ± 0.08) × 10^2^	< MDC	(1.04 ± 0.02) × 10^4^	(5.39 ± 0.11) × 10^4^	< MDC	(4.80 ± 0.01) × 10^1^	< MDC	(4.15 ± 0.08) × 10^3^	(1.38 ± 0.02) × 10^3^	(1.43 ± 0.03) × 10^3^	(1.41 ± 0.03) × 10^3^	(4.22 ± 0.09) × 10^3^
14	(3.28 ± 0.07) × 10^2^	(4.81 ± 0.01) × 10^2^	< MDC	< MDC	(5.96 ± 0.12) × 10^2^	< MDC	(1.29 ± 0.03) × 10^2^	< MDC	(4.15 ± 0.08) × 10^6^	(1.13 ± 0.02) × 10^2^	(1.16 ± 0.02) × 10^2^	< MDC	(2.29 ± 0.04) × 10^2^
15	< MDC	(1.63 ± 0.38) × 10^4^	< MDC	< MDC	(3.23 ± 0.06) × 10^2^	< MDC	< MDC	< MDC	< MDC	< MDC	< MDC	< MDC	< MDC
16	(2.41 ± 0.05) × 10^3^	(1.67 ± 0.03) × 10^2^	< MDC	< MDC	(4.23 ± 0.08) × 10^2^	< MDC	(9.60 ± 0.2) × 10^1^	< MDC	(4.73 ± 0.09) × 10^5^	(1.13 ± 0.02) × 10^2^	(1.18 ± 0.02) × 10^2^	< MDC	(2.31 ± 0.04) × 10^2^
17	(1.82 ± 0.04) × 10^2^	(2.06 ± 0.04) × 10^4^	< MDC	(1.20 ± 0.02) × 10^2^	(1.12 ± 0.02) × 10^3^	< MDC	< MDC	< MDC	(4.14 ± 0.08) × 10^4^	(2.87 ± 0.06) × 10^2^	(3.15 ± 0.06) × 10^2^	< MDC	(6.02 ± 0.12) × 10^2^
18	(2.54 ± 0.05) × 10^3^	(5.79 ± 0.12) × 10^2^	< MDC	< MDC	(5.15 ± 0.10) × 10^2^	< MDC	(2.17 ± 0.04) × 10^2^	< MDC	(4.81 ± 0.10) × 10^5^	(1.17 ± 0.02) × 10^2^	(1.22 ± 0.02) × 10^2^	< MDC	(2.39 ± 0.04) × 10^2^
19	(3.25 ± 0.07) × 10^2^	(3.91 ± 0.08) × 10^2^	< MDC	(7.97 ± 0.16) × 10^3^	(5.33 ± 0.11) × 10^4^	< MDC	(3.4 ± 0.1) × 10^1^	< MDC	(4.27 ± 0.09) × 10^3^	(1.39 ± 0.03) × 10^3^	(1.47 ± 0.03) × 10^3^	(1.42 ± 0.03) × 10^3^	(4.27 ± 0.08) × 10^3^
20	< MDC	(6.68 ± 0.13) × 10^2^	< MDC	< MDC	< MDC	(7.02 ± 0.14) × 10^3^	(4.2 ± 0.1) × 10^1^	< MDC	< MDC	(5.33 ± 0.11) × 10^3^	(5.10 ± 0.10) × 10^2^	(5.20 ± 0.10) × 10^2^	(1.56 ± 0.03) × 10^3^
21	(1.21 ± 0.02) × 10^3^	(1.40 ± 0.03) × 10^3^	(1.96 ± 0.04) × 10^2^	(5.91 ± 0.12) × 10^3^	(8.11 ± 0.16) × 10^4^	< MDC	(1.40 ± 0.03) × 10^2^	< MDC	(2.92 ± 0.06) × 10^4^	(2.90 ± 0.06) × 10^3^	(3.04 ± 0.06) × 10^3^	(2.98 ± 0.06) × 10^3^	(8.92 ± 0.18) × 10^3^
22	(2.22 ± 0.04) × 10^2^	(4.03 ± 0.08) × 10^2^	< MDC	(5,99 ± 0.12) × 10^3^	(5.09 ± 0.10) × 10^4^	< MDC	(2.79 ± 0.06) × 10^2^	< MDC	(3.97 ± 0.08) × 10^3^	(1.35 ± 0.03) × 10^3^	(1.39 ± 0.03) × 10^3^	(1.38 ± 0.03) × 10^3^	(4.12 ± 0.09) × 10^3^
23	(2.55 ± 0.05) × 10^2^	(4.25 ± 0.09) × 10^3^	< MDC	(3.99 ± 0.08) × 10^2^	(3.65 ± 0.07) × 10^3^	< MDC	< MDC	< MDC	(5.43 ± 0.11) × 10^3^	(2.43 ± 0.05) × 10^2^	(2.66 ± 0.05) × 10^2^	< MDC	(5.09 ± 0.10) × 10^2^
24	(2.25 ± 0.05) × 10^2^	(1.76 ± 0.04) × 10^3^	< MDC	(5.89 ± 0.12) × 10^3^	(6.30 ± 0.12) × 10^4^	< MDC	< MDC	< MDC	(8.94 ± 0.18) × 10^3^	(2.29 ± 0.05) × 10^3^	(2.40 ± 0.05) × 10^3^	(2.35 ± 0.05) × 10^3^	(7.03 ± 0.15) × 10^3^
25	(1.82 ± 0.04) × 10^2^	(2.06 ± 0.04) × 10^4^	< MDC	(1.20 ± 0.02) × 10^2^	(1.12 ± 0.02) × 10^3^	< MDC	< MDC	< MDC	(4.14 ± 0.08) × 10^4^	(2.87 ± 0.06) × 10^2^	(3.15 ± 0.06) × 10^2^	< MDC	(6.02 ± 0.12) × 10^2^
26	(1.82 ± 0.04) × 10^2^	(2.06 ± 0.04) × 10^4^	< MDC	(1.20 ± 0.02) × 10^2^	(1.12 ± 0.02) × 10^3^	< MDC	< MDC	< MDC	(4.14 ± 0.08) × 10^4^	(2.87 ± 0.06) × 10^2^	(3.15 ± 0.06) × 10^2^	< MDC	(6.02 ± 0.12) × 10^2^
27	< MDC	(6.68 ± 0.13) × 10^2^	< MDC	< MDC	< MDC	(7.02 ± 0.14) × 10^3^	(4.20 ± 0.10) × 10^1^	< MDC	< MDC	(5.33 ± 0.11) × 10^2^	(5.10 ± 0.10) × 10^2^	(5.20 ± 0.10) × 10^2^	(1.56 ± 0.03) × 10^3^
28	< MDC	< MDC	< MDC	< MDC	< MDC	< MDC	< MDC	< MDC	< MDC	< MDC	< MDC	< MDC	< MDC
29	< MDC	< MDC	< MDC	< MDC	< MDC	< MDC	(4.20 ± 0.10) × 10^1^	< MDC	< MDC	< MDC	< MDC	< MDC	< MDC
30	< MDC	< MDC	< MDC	< MDC	< MDC	< MDC	(4.20 ± 0.10) × 10^1^	< MDC	< MDC	< MDC	< MDC	< MDC	< MDC
MDC	(1.51 ± 0.03) × 10^2^	57 ± 1	(1.54 ± 0.03) × 10^2^	(7.10 ± 0.10) × 10^1^	(9.30 ± 0.2) × 10^1^	(1.10 ± 0.05) × 10^1^	(2.00 ± 0.05) × 10^0^	-	(3.30 ± 0.66) × 10^1^	(8.50 ± 0.20) × 10^1^	(8.60 ± 0.20) × 10^1^	(4.11 ± 0.08) × 10^2^	(8.50 ± 0.20) 10^1^
Reference^a^	5.00 × 10^4^	1.00 × 10^5^	-	2.00 × 10^4^	2.00 × 10^6^	1.00 × 10^4^	10.00 × 10^4^	3.00 × 10^3^	7.00 × 10^5^	-	-	-	1.00 × 10^4^
Min	(1.82 ± 0.04) × 10^2^	(1.67 ± 0.03) × 10^2^	(1.62 ± 0.03) × 10^2^	(9.0 ± 0.2) × 10^1^	(1.43 ± 0.03) × 10^2^	(6.23 ± 0.13) × 10^2^	(4.20 ± 0.10) × 10^1^	-	(3.97 ± 0.08) × 10^3^	(8.90 ± 0.20) × 10^1^	(9.00 ± 0.20) × 10^1^	(3.57 ± 0.07) × 10^2^	(8.70 ± 0.20) × 10^1^
Max	(2.79 ± 0.06) × 10^3^	(4.63 ± 0.09) × 10^5^	(3.02 ± 0.06) × 10^3^	(1.04 ± 0.02) × 10^4^	(8.50 ± 0.17) × 10^4^	(3.42 ± 0.07) × 10^4^	(2.79 ± 0.06) × 10^2^	-	(4.16 ± 0.08) × 10^6^	(3.69 ± 0.08) × 10^3^	(3.83 ± 0.08) × 10^3^	(3.77 ± 0.08) × 10^3^	(1.06 ± 0.02) × 10^4^
Mean	(6.61 ± 0.13) × 10^2^	(2.61 ± 0.05) × 10^4^	(1.18 ± 0.02) × 10^2^	(4.25 ± 0.09) × 10^2^	(3.80 ± 0.08) × 10^3^	(1.26 ± 0.03) × 10^4^	(7.60 ± 0.20) × 10^1^	-	(6.25 ± 0.13) × 10^4^	(6.04 ± 0.12) × 10^2^	(6.12 ± 0.12) × 10^2^	(6.06 ± 0.12) × 10^2^	(1.95 ± 0.06) × 10^3^

^a^Reference value according to the Ministry of Health of Indonesia regulations and WHO.

## Radiation exposure measurements

### Indoor-to-outdoor dose-rate ratio

**Figure 1:**
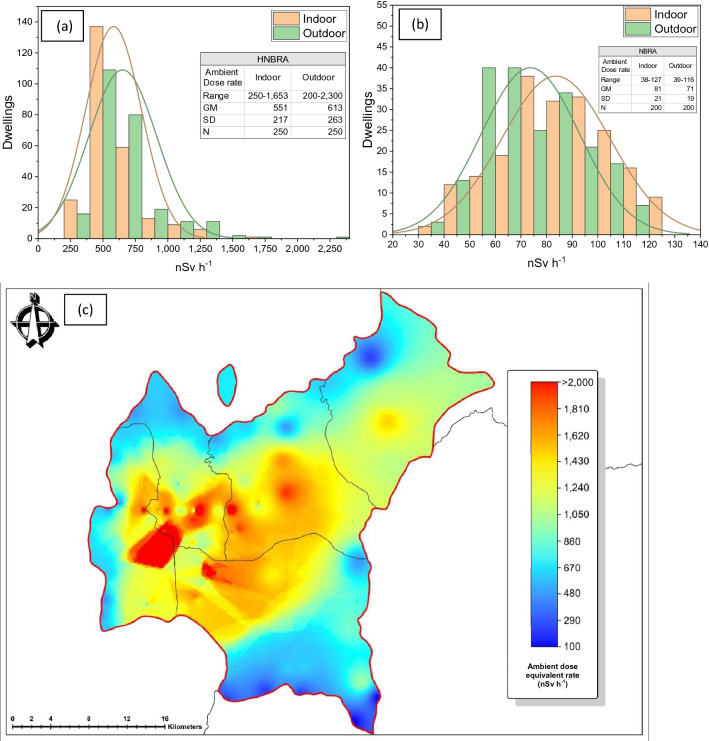
Indoor and outdoor ambient dose equivalent rate distributions in the: (**a**) HNBRA and (**b**) the NBRA. (**c**) Ambient dose equivalent dose-rate map of Mamuju region. Maps were created in MAPINFO PROFESSIONAL version 10.5 (https://www.precisely.com/product/precisely-mapinfo/mapinfo-pro) using map data derived from the National Mapping Agency of Indonesia (https://tanahair.indonesia.go.id/portal-web).

The measurement results of ambient dose equivalent rates in the HNBRA (Fig. 1a) for residential houses indoors had a geometric mean of 551 nSv h^−1^ with a range of 250–1653 nSv h^−1^; the rates outdoors had a geometric mean of 613 nSv h^−1^ with a range of 200–2300 nSv h^−1^. The measurement results of ambient dose equivalent rates in the normal background radiation area (NBRA) or the control area (Fig. 1b) for indoors had a geometric mean of 81 nSv h^−1^ with a range of 38–127 nSv h^−1^; the rates outdoors had a geometric mean of 71 nSv h^−1^ with a range of 39–116 nSv h^−1^. Figure 1c is the ambient dose equivalent rate map in Mamuju drawn using the collected outdoor ambient gamma doses. The distribution of radiation exposure in the Mamuju HNBRA is wide with several high exposure hotspots (outliers) found. Indoor-to-outdoor dose-rate ratio in the HNBRA had the range of 0.57–1.51 with geometric mean 0.90 and for control area had the range of 0.66–2.02 with geometric mean 1.13.

### Activity concentrations from foodstuffs, drinking water, radon and thoron

The respective average activity concentrations for ^226^Ra, ^232^Th and ^40^K in foodstuffs were: rice, 0.4 Bq kg^−1^, 6.2 Bq kg^−1^, and 87.9 Bq kg^−1^; meat/fish/vegetables, 36 Bq kg^−1^, 57.1 Bq kg^−1^, and 971.5 Bq kg^−1^; fruits, 7.4 Bq kg^−1^, 7.4 Bq kg^−1^, and 390.8 Bq kg^−1^. Details regarding radioactivity in foodstuffs can be found in Supplementary Table S2.

**Figure 2:**
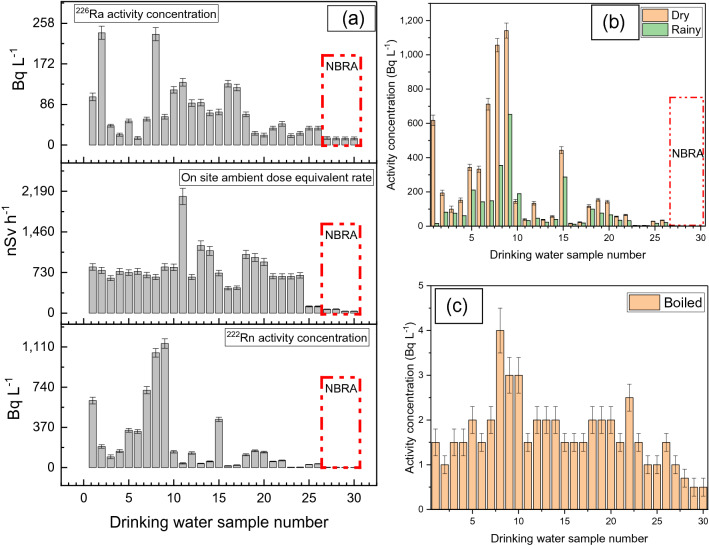
(**a**) Radium and radon activity concentration measurement results in drinking water; and ambient dose equivalent rate measurements. (**b**) Radon measurement results in drinking water for rainy and dry seasons. (**c**) Radon measurement results in drinking water after boiling. The graphs in (**a**) include results of the present research and the literature^21^. NBRA refer to the normal background radiation area or control area.

From radioactivity measurements in drinking water samples, the ^226^Ra concentration ranged from 14 to 238 mBq L^−1^ (Fig. 2a). The highest drinking water concentration of ^226^Ra was obtained in Botteng Village. For the control area and also for bottled water, the observed concentrations were small, around 14 mBq L^−1^. The measured radon concentrations in drinking water (Fig. 2b) ranged from 1 to 1141 Bq L^−1^ in the dry season and 1–652 Bq L^−1^ in the rainy season. Figure 2c shows the radon concentration in water after a boiling process.

**Figure 3:**
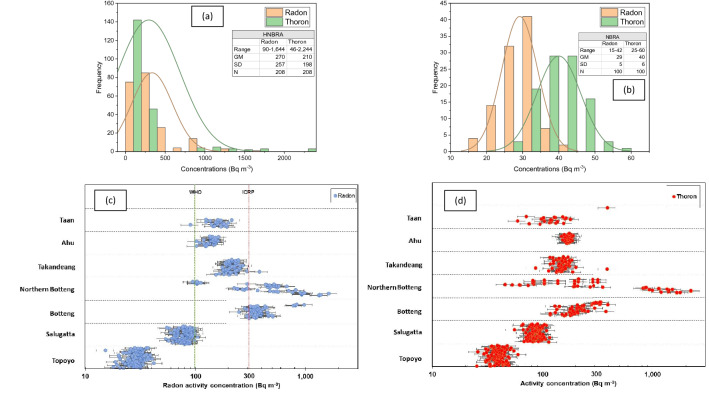
Annual average radon and thoron concentrations in houses. (**a**) Distributions of ^222^Rn and ^220^Rn in the HNBRA. (**b**) Distributions of ^222^Rn and ^220^Rn in the NBRA. (**c**) Annual average ^222^Rn concentration in the study villages and control area. (**d**) Annual average ^220^Rn concentration in the study villages and control area. In (**c**,**d**) the horizontal black lines are the uncertainties. WHO^26^ and ICRP^27^ reference levels for indoor ^222^Rn activity concentration are 100 and 300 Bq m^−3^, respectively.

Radon concentrations in the HNBRA had a geometric mean of 270 Bq m^−3^ with a range of 90–1644 Bq m^−3^ while ^220^Rn concentrations had a geometric mean of 210 Bq m^−3^ with a range of 46–2244 Bq m^−3^ (Fig. 3a). The ^222^Rn and ^220^Rn concentrations in the NBRA (control area) are plotted in Fig. 3b. Figure 3c,d show the radon concentrations and thoron concentrations in each village of the HNBRA and the NBRA. The equilibrium equivalent thoron concentration (EETC) in the HNBRA obtained with the thoron progeny monitor had a range of 2–42 Bq m^−3^ with the geometric mean of 11 Bq m^−3^. For the NBRA the EETC had a range of 0.4–4 Bq m^−3^ with geometric mean of 1.9 Bq m^−3^.

## Discussion

### Characteristic exposure and source identification

The presently accepted worldwide average activity concentrations of ^40^K, ^238^U, and ^232^Th nuclides are 412, 32, and 45 Bq kg^−1^, respectively^2^. We compared our obtained average activity concentrations with these worldwide average values. The concentrations of natural radionuclides in soil samples in the Mamuju region are very high, as shown in Supplementary Figure S2 and Table S1. The concentration of ^238^U is 23–30 times greater than the world average, and that of ^232^Th is 27–60 times greater. The Mamuju region is unique because it has a high concentration of both ^238^U and ^232^Th natural radionuclides.

From the soil radioactivity results (Supplementary Figure S2), we can identify several characteristics of this HNBRA: dominant areas for ^238^U include Botteng and Taan Villages; and dominant areas for ^232^Th include Takandeang, Northern Botteng, and Ahu. The main sources of external radiation are uranium and thorium. Supplementary Figure S3 shows the correlation analysis between radioactivity (^238^U, ^232^Th and ^40^K) in the soil with the ambient dose-rate. The negative relationship of ^40^K occurs because the ^238^U and ^232^Th contents are high so that the potassium concentration is not significant. These results reinforce the results of a previous study using a scintillation detector^16^.

The external exposure predicted from the indoor-to-outdoor dose-rate ratio (range 0.57–1.51; geometric mean 0.90) in the HNBRA is higher than that indoors. This indicates that the radiation originates from outside the house because inside, there is a shielding effect. Most houses in the HNBRA have wood walls and coated cement floors. In a low radiation area, the radiation exposure inside the house is slightly higher than outdoors where the indoor-to-outdoor dose-rate ratio ranges between 0.66 and 2.02 (geometric mean, 1.13). This may be the influence of the building materials used. In Topoyo Village (NBRA) and Salugatta Village (a medium radiation area), the houses are made with brick walls and ceramic floors. This difference in building types from the HNBRA occurs because Salugatta and Topoyo are transmigration areas, so they have a slightly different lifestyle between other villages studied in the HNBRA. The indoor-to-outdoor dose-rate ratio in Mamuju is slightly more homogeneous comparing with a study in Kerala, India where the ratio ranged between 0.08 and 1.20^7^.

### Annual mean effective dose estimation

The annual effective dose is determined from the accumulated external and internal doses received by the public. For external dose contribution, we note that the estimated dose excludes external exposure due to cosmic radiation. External radiation measurements can be made using a personal dosimeter or measuring indoor and outdoor ambient dose with a survey meter. Measurement of indoor-to-outdoor dose-rate with a survey meter has limitations because it must be multiplied by the occupancy factor, while residents often change places. Based on this fact we used a personal dosimeter to determine external exposure, referred to in UNSCEAR^2^ as personal dose equivalent. The personal dosimeter measures the integration of external radiation so no conversion factors are required.

The individual external effective dose obtained using the personal dosimeter in the high radiation area ranges from 1.9 to 14.3 mSv year^−1^ with a geometric mean of 4.7 mSv year^−1^. For the NBRA (control area), the individual external effective dose ranges from 0.3 to 0.9 mSv year^−1^ with a geometric mean of 0.5 mSv year^−1^. For the medium radiation area in the HNBRA, the individual external effective dose ranges from 0.5 to 1.0 mSv year^−1^ with geometric mean of 0.75 mSv year^−1^.

Most Mamuju residents consume natural drinking water and foods they have grown or produced themselves. Almost all foodstuffs in Mamuju have a concentration activity that exceeds the reference value of the IAEA, although they do not exceed a value of about 1 mSv annually. The reference values for meat, fruits and leafy vegetables respectively are 0.015 Bq kg^−1^, 0.030 Bq kg^−1^, and 0.050 Bq kg^−1^ for ^226^Ra and 0.001 Bq kg^−1^, 0.001 Bq kg^−1^, 0.015 Bq kg^−1^ for ^232^Th^28^. Contributions of rice, meat + vegetables and fruits to annual effective dose, respectively, are 0.01–0.14 mSv (geometric mean, 0.04 mSv), 0.01–2.51 mSv (geometric mean 0.54 mSv) and 0.01–0.1 mSv (geometric mean 0.05 mSv). Among the radioactivity results obtained in foodstuffs, spinach is especially high. The presence of radionuclides in food may be a result of root uptake from the soil, direct deposition from the atmosphere onto crops or transfer through aquatic pathways. Regarding this, it is necessary to carry out further research on the transfer of factors from soil to these foodstuffs.

The results obtained from the measurement of radioactivity in drinking water in Mamuju are shown in Fig. 2a. Samples from the HNBRA have higher activity concentration than those from the NBRA. The highest radon activity concentration in drinking water is 1141 Bq L^−1^, and in the rainy season the values are smaller than in the dry season (summer) (Fig. 2b). The annual effective doses from radon in drinking water are between 0.01 and 2.33 mSv (geometric mean 0.02 mSv). Several drinking water samples have radon concentrations exceeding 100 Bq L^−1^. The United States Environmental Protection Agency (USEPA) proposed a maximum contaminant level (MCL) for radon in drinking water of 11 Bq L^−1^ and an alternative maximum contaminant level (AMCL) of 148 Bq L^−1^^29,30^. On the other hand, the European Union (EU) recommended that the reference level for ^222^Rn in drinking water is 100 Bq L^−1^^31^. Additionally, remedial action without further consideration is justified in all EU countries if radon concentration in drinking water exceeds 1000 Bq L^−1^^31^. However, the radon concentration in drinking water will decrease after boiling, as shown in Fig. 2c. For this reason, radon released when boiling water must be considered, as it will increase the concentration of radon in the air. This released atmospheric radon poses a larger public health risk than radon that enters through ingestion. The radium contribution in drinking water to the annual effective dose is small and it ranges from 0.01 to 0.04 mSv (geometric mean 0.01 mSv).

For heavy metals in drinking water, some samples have values above the allowable limit according to the Indonesian Ministry of Health regulations^33^ and WHO guidelines for water quality^24,25,32^. Most drinking water in Mamuju contains higher concentrations of heavy metals than bottled drinking water. Heavy metal contents might be due to environmental influences such as being in a HNBRA, which has high radioactivity in soil. The natural decay process of radionuclides can lead to heavy metals, in particular Pb.

The WHO proposed a reference level of 100 Bq m^−3^ to minimize health hazards due to indoor radon exposure^26^. Additionally, the report stated that if the reference level of 100 Bq m^−3^ cannot be reached, the chosen reference level should not exceed 300 Bq m^−3^ according to ICRP recommendations^27^. Around 99% of the houses in the HNBRA have ^222^Rn activity concentrations exceeding the WHO reference level and 40% of the houses exceed the ICRP recommendations. The highest ^222^Rn and ^220^Rn concentrations are in Tande-Tande and Northern Botteng Villages, with a concentration of 1644 Bq m^−3^ (presented later in Fig. 5). Annual effective doses from radon in the HNBRA are between 4 and 78 mSv (geometric mean 13 mSv). This value is extremely high and correlates with concentrations of ^238^U and ^232^Th in soil samples in the area, which are parents of ^222^Rn and ^220^Rn.

**Figure 4:**
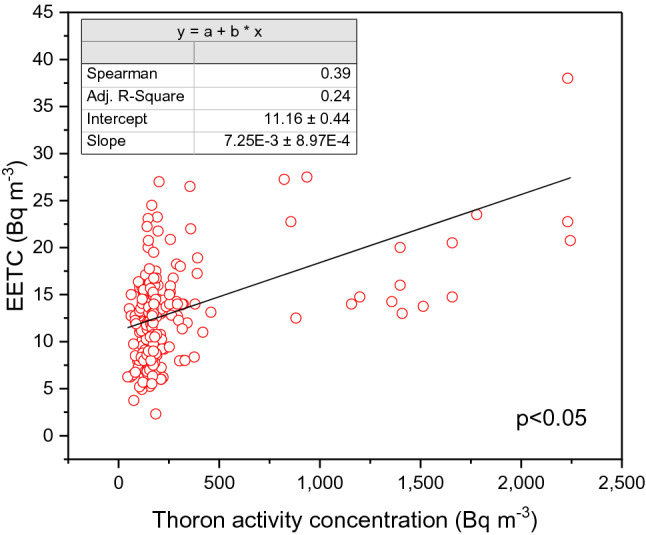
Correlation between thoron concentration and EETC.

According to the properties of ^220^Rn, which has a short half-life of about 56 s, this can cause a problem for the measurement because the ^220^Rn concentration will depend on the distance from the radionuclide source to the detector. To solve this problem, the thoron progeny concentration was directly measured. The thoron progeny monitor was used to obtain the EETC. Figure 4 shows the correlation between EETC and thoron concentration. Annual effective doses from thoron are between 3 and 30 mSv (geometric mean 9 mSv).

In the case of radiation exposure from background radiation and technologically enhanced naturally occurring radioactive material (TENORM), the ICRP has classified it as the "existing exposure" situation^33^. In the radiological protection system, the ICRP stated that the reference level used in conjunction with the optimization of protection to restrict individual dose due to "existing exposure" is between 1 and 20 mSv.

**Figure 5:**
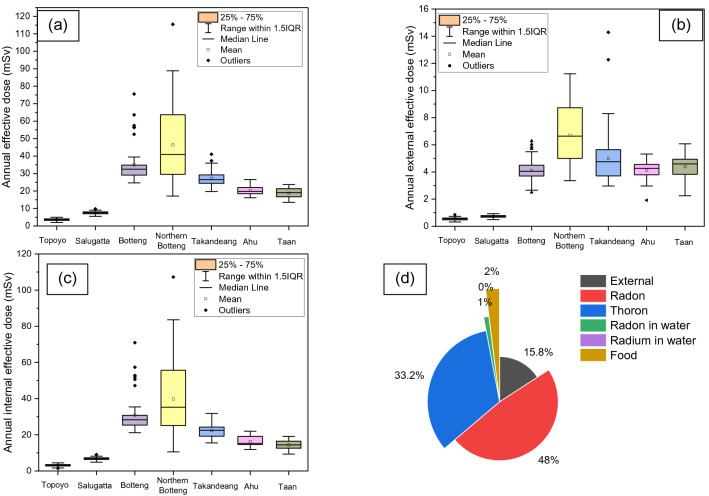
Comparison of (**a**) annual internal effective dose and (**b**) annual external effective dose in the study villages and control area to the annual effective dose. (**c**) The annual effective dose. (**d**) Pie chart showing the contribution of each dose to the annual effective dose.

The estimated annual effective dose in Mamuju HNBRA is shown in Fig. 5. The annual effective dose (geometric mean) from Topoyo, Salugatta, Botteng, Northern Botteng, Takandeang, Ahu, and Taan villages respectively are 2–5 (3.6) mSv, 5–10 (7.5) mSv, 25–75 (33.9) mSv, 17–115 (41.6) mSv, 20–41 (27) mSv, 16–27 (20.1) mSv, and 13–24 (18.5) mSv. For the whole HNBRA, the average annual effective dose is 32 mSv and the geometric mean is 29.7 mSv. This value exceeds the global mean of 2.4 mSv and also the highest value exceeds the upper value of the reference level for existing exposure situations. The largest contribution to the annual effective dose comes from radon and thoron gases. Radon gas contributes 48% to the annual effective dose, while thoron gas, external dose, foodstuffs and drinking water contribute 33.2%, 15.8%, 1%, and 2%, respectively.

Dose data from the present study HNBRA and other HNBRAs in the world are listed in Table 3. We note that the estimated annual effective dose of radon and thoron in this study used the dose coefficient of ICRP 137, which is slightly higher than the previous ICRP publication. We can see that Mamuju is a unique HNBRA because it has large external and internal doses. Besides that, the landscape in Mamuju is still natural and there is no mining; the radiation exposure distribution is wide and there are many residents.

**Table 3:** A comparison of doses and dose coefficients of radon and thoron between the present study of the Mamuju HNBRA and other previously studied HNBRAs.

Country	HNBRA	Area characteristic	Average annual internal dose (mSv)	Average annual external dose (mSv)	Average annual effective dose (mSv)	Dose coefficient or dose conversion factor of radon (mSv (Bq h m^−3^)^−1^)		Dose coefficient or dose conversion factor of thoron (mSv (Bq h m^−3^)^−1^)	Reference
Brazil	Guarapari	Monazite sands	–	–	7	–		–	^3,34^
China	Yangjiang	Monazite particles	4.1	2.1	6.4	9 × 10^–6^		4 × 10^–5^	^3,8,34^
India	Kerala	Monazite sands	2.4	4.5	6.9	9 × 10^–6^		4 × 10^–5^	^7,9,34^
Iran	Ramsar	Sulfurous hot springs and ^226^Ra	4.2	6	10.2	9 × 10^–6^		–	^3^
Present study	Mamuju	Volcanic rocks and basalt rock type containing ^232^Th and ^238^U	26.9	5	32	1.7 × 10^–5^		1.07 × 10^–4^	Present study
World average			–	0.48	2.4	–		–	^2^

### The implication of the HNBRA to human health and its use as an epidemiological study area related to chronic low-dose-rate radiation from environmental sources

Residents in Mamuju are completely dependent on nature for their daily activities which include working in vegetable and fruit gardens and fetching water for drinking from wells. Almost all of their time is spent in a high radiation area. The HNBRA can lead to environmental pollution caused by radiation and heavy metals from radionuclide decay that eventually enter the human body. The activity concentration data presented in this paper have high values, even exceeding the values for radiation workers, and they are of particular concern from the viewpoint of public health.

Radon and thoron gas are the radionuclides which make the most significant contribution to the annual dose received by humans, especially in the HNBRA. Based on the radioactivity characteristics from soil samples in Mamuju that have ^238^U and ^232^Th radionuclides, measurements of ^222^Rn, ^220^Rn, and their progeny are essential. The monitoring results of ^222^Rn and ^220^Rn are also influenced by the characteristics of the area, which shows that the dose contribution comes from NORM contained in the area soil. Many factors affect the concentration of radon and thoron in residential dwellings. The most influential factor is air ventilation^35^. Radon gas diffuses easily and disappears from dwellings that have proper air ventilation, but based on the characteristics of natural exposure and radioactivity in the soil of the Mamuju HNBRA, it will be challenging to reduce the ^222^Rn and ^220^Rn concentration there.

The high internal doses of radon can cause human health problems, especially in the respiratory tract. Moreover, combined exposure to radon and tobacco smoke may further increase lung cancer risk. The alpha particles emitted by radon and its progeny can directly attack genomic DNA and cause mainly double-strand breaks in DNA^36–38^. Also, overproduction of reactive oxygen species in the lungs caused by persistent radon exposure may cause oxidative stress, leading to inflammation of pulmonary, tissue damage, and eventually to chronic diseases of the lung such as chronic obstructive pulmonary disease (COPD), pulmonary fibrosis, and lung cancer^36^. Strong and complementary risk evidence for lung cancer from cumulative exposure to radon and its progeny through inhalation has been determined from studies of occupational exposures of uranium miners and residential exposures of the public^4^. According to the WHO Handbook^26^, the risk of lung cancer increases by 16% per 100 Bq m^−3^ increase in radon concentration. Zhang et al.^39^ reported on a meta-analysis of case–control studies on residential radon and lung cancer risk that showed for every 100 Bq m^−3^, an increase in radon exposure was associated with a significant 7% increase in lung cancer risk. Li et al.^40^ reported radon exposure was associated with risk increases in lung cancer, small-cell lung carcinoma and adenocarcinoma by 11%, 19% and 13%, respectively. Therefore, the dose–response relationship is linear, i.e., the risk of lung cancer is proportional to radon exposure. As well, several heavy metals can induce cancer (e.g., arsenic, As) or non-cancer disease (e.g., mercury, Hg) risks in humans^12^.

Conducting nested case–control studies, with dose assessment on an individual level and collection of individual information on known as well as other possible risk factors for the diseases of interest, will be a useful tool for the evaluation of any potential health risks from low-level chronic radiation exposures^4^. From public health data, upper respiratory tract infection, gastritis, and hypertension are the dominant illnesses in Mamuju (see Fig. 7). If we consider the internal dose received by the population of Mamuju, it is necessary to study the situation further epidemiologically.

Epidemiological studies of populations in HNBRAs exposed to radiation delivered at low dose-rates over long periods leading to cumulative doses up to several hundred millisieverts offer an opportunity to investigate the health effects associated with chronic low-dose-rate radiation exposure. The epidemiological studies of populations exposed to environmental sources of radiation offer an opportunity to obtain risk estimates for the induction of cancer from low-dose-rate radiation exposure up to a cumulative dose of 0.5 Sv or more^4,41^. The lifetime cumulative dose is calculated by multiplying 70 years by the annual effective dose. Based on the data presented in this paper, the lifetime cumulative dose calculation suggests that the residents in Mamuju could be exposed to 2.2 Sv on average (range 1.2–8.1 Sv) which is much higher than the average doses for atomic bomb survivors (average dose: 200 mSv) and for most other exposed populations. In the case of the atomic bombs survivors, they were received acute exposure, whereas residents in the HNBRA have being exposed to chronic radiation. Therefore, we need to continue studies on the differences in human effects due to acute and chronic exposures. Epidemiological studies in several regions of the world (Ramsar, Yangjiang, Kerala and Guarapari) reported no correlation between radiation exposures in the HNBRA and cancer rate or mortality^42–45^. The same description was found in the 2017 UNSCEAR report^4^, i.e. the effect of low dose-rates on health and cancer risks after exposure to ionizing radiation is, as yet, unclear. Based on this situation, Mamuju is a potential area for epidemiological study of health risks (cancer and non-cancerous) due to the chronic low-dose-rate radiation from environmental sources.

## Material and methods

### Study area and population

We used a cross-sectional study method with cluster sampling areas. For natural radioactivity measurements, soil and food samples were analysed in the National Nuclear Energy Agency of Indonesia, Center for Technology of Radiation Safety and Metrology, National Environmental Safety Laboratory, which is accredited to ISO17025 and a member of the IAEA-Analytical Laboratories for the Measurement of Environmental Radioactivity (ALMERA network laboratories). Other analyses were performed at Hirosaki University, Institute of Radiation Emergency Medicine, Japan. Table 4 summarizes the radionuclides and elements analysed and the analytical techniques used for their determination.

**Table 4:** Summary of methods.

Sample	Radionuclide or elements	Purpose of measurement	Preparation	Analytical technique	Number of measurements
External radiation	Gamma-ray	Ambient dose equivalent rate, H*(10) (indoor-to-outdoor dose-rate ratio)	1 m above the soil surface	Direct counting on survey meter	450
	Gamma-ray	Personal dose equivalent, H_p_(10) (external exposure assessment)	Used with personal dosimeter	Direct counting on OSL dosimeter and worn by residents	120
Soil	^238^U, ^232^Th, ^235^U, and ^40^K	Original sources for gamma-rays, radon and thoron	Dry, sieve and store in standard Marinelli beakers	30-day wait for secular equilibrium before HPGe measurement	30
Water	Cu, Cr, Cd, Mn, Co, As, Se, Ba, ^206^Pb, ^207^Pb, ^208^Pb and total Pb	ingestion, heavy metal	Acidify	ICP-MS QQQ	30
	^222^Rn	Internal dose, ingestion	250 mL H_2_O kit bottle, rainy season and dry season measurements	Bubble method, direct counting on RAD7 radon active monitor with H_2_O kit	30
	^226^Ra	Internal dose, ingestion	Acidify, 10:10 mL water/Ultima Gold uLLT	30-day wait for secular equilibrium before liquid scintillation counting	30
	F^−^, Cl^−^, NO_3_^−^, Na^+^, and Ca^2+^	Water quality	Direct	Dionex ion chromatography system	30
	Temperature, pH, TDS	Water quality	Direct	Digital thermometer, pH meter and EC meter	30
Foodstuffs	^226^Ra, ^232^Th, ^40^K	Internal dose, ingestion	Freeze dry and store in standard Marinelli beakers	30-day wait for secular equilibrium before HPGe measurement	30
Air	^222^Rn, ^220^Rn and ^212^Po	Internal dose, Inhalation	1-year measurement with monitor replacement at 3-month intervals	Passive radon monitor, SSNTDs (CR-39) RADUET	408

**Figure 6:**
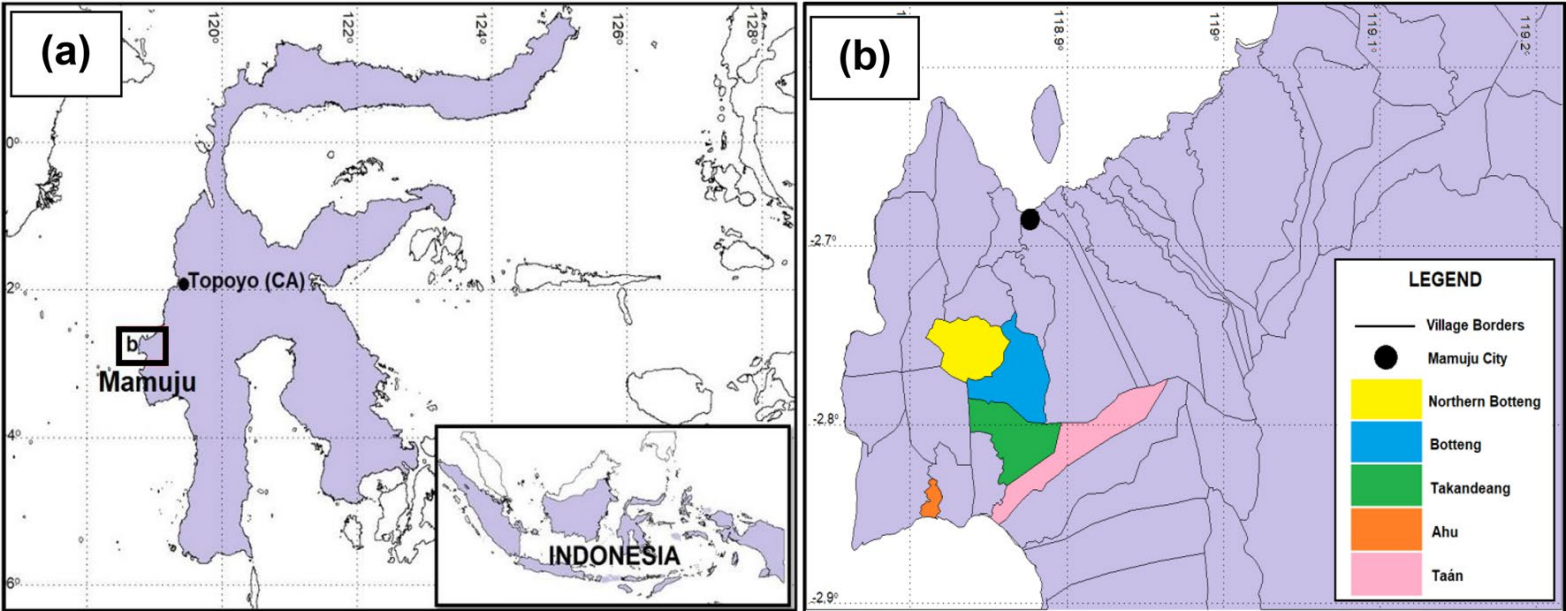
Location maps. (**a**) Sulawesi Island, including Mamuju region which was the study HNBRA and Topoyo Village which was the study NBRA. (**b**) Villages of the study in Mamuju region. HNBRA refer to the high natural background radiation area and control area (CA) refers to the normal background radiation area (NBRA) or control area. Maps were created in MAPINFO PROFESSIONAL version 10.5 (https://www.precisely.com/product/precisely-mapinfo/mapinfo-pro) using map data derived from the National Mapping Agency of Indonesia (https://tanahair.indonesia.go.id/portal-web).

**Figure 7:**
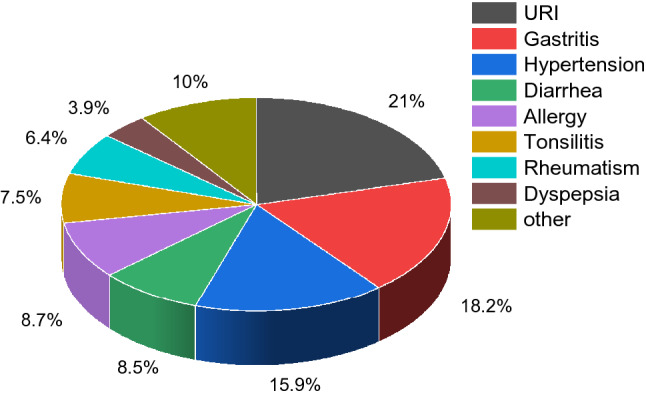
Pie chart summarizing the main diseases suffered by Mamuju region residents^46^. URI stands for upper respiratory tract infection.

The study involved villages in the two cities of Mamuju and Mamuju Tengah, located in West Sulawesi Province on Sulawesi Island. Location maps of the island and the villages are shown in Fig. 6. This study was conducted from November 2018 to March 2020. West Sulawesi Province had a population of 432,000 people in 2018 on an area of 5406 km^2^. The Mamuju region has a tropical climate with two seasons, the dry season from April to September and the rainy season from October to March. The landscape is still natural, with rivers and mountain topography across almost all sub-districts and there is no mining activity^46^. Mamuju City had 286,389 people in the agricultural sector working as farmers, representing 62.2% of the population; and the primary agricultural product is cacao, which is exported to foreign countries. Most of the houses in Mamuju City are of wood (around 57.6%); but 40.8% have brick walls among which 39.7% have a cement floor and 31.6% a wooden floor. The most common diseases suffered by residents (Fig. 7) are upper respiratory tract infectionsURI, with 21,070 cases and gastritis with 18,177 cases^46^ and life expectancy is around 67.2 years.

### Lifestyle and consumption behavior questionnaire survey

We conducted a short questionnaire survey of residents to determine their lifestyle and consumption behaviors. All questionnaire surveys were performed in accordance with relevant guidelines and regulations; 408 families covering the whole study area were surveyed and we confirmed that informed consent was obtained from all subjects and/or their legal guardians. The study itself was approved by the Committee of Medical Ethics of Hirosaki University Graduate School of Medicine (2018-089, Hirosaki, Japan).

Based on previous results of the car-borne survey^16^, it was found that several areas in Mamuju have significant gamma exposure, including Northern Botteng, Botteng, Takandeang, Ahu and Taan Villages (high radiation area villages). For the study control area, a NBRA was selected, Topoyo Village in Mamuju Tengah City about 120 km from Mamuju City. Between the high radiation area villages and NBRA control area, Salugatta Village was a medium area having an ambient dose-rate ranging from 100 to 200 nSv h^−1^. The residential populations and the surveyed populations of each village are shown in Table 5.

**Table 5:** Resident populations and surveyed populations in the study area.

Village name	Population				Surveyed population				
	Number of households	Male	Female	Total	Number of households	Male	Female	Child	Total
Botteng	450	1029	1012	2041	55	95	94	16	205
Northern Botteng	562	1265	1239	2504	51	100	77	62	239
Takandeang	524	1172	1128	2300	51	140	160	14	314
Ahu	316	720	749	1469	30	66	61	44	171
Taan	808	2027	1962	3989	22	40	39	33	112
Salugatta	783	1595	1567	3162	100	192	182	62	436
Topoyo (control area)	1516	3802	3583	7385	100	216	208	86	510
Total	–	145,479	140,910	286,389	408	849	821	317	1987

### Natural radionuclide activity concentrations in soil samples

A total of 30 soil samples of 1 kg each were collected from 30 locations including 24 samples in the HNBRA and 6 samples in the control area. The location of sample selection was carried out randomly by considering the number of hamlets in a village and the presence of residents in the village because some hamlets were no longer inhabited. Detailed information on the location is shown in the Supplementary information. The soil samples were crushed and sieved, and placed in sealed standard Marinelli beakers. Their decay products were allowed to reach secular equilibrium (30 days) and then they were measured using a high purity germanium (HPGe) detector.

The HPGe detector was calibrated using a standard source (NIST, USA) with the same geometry as the Marinelli beakers for samples. A p-type HPGe detector (GEM 60-5, ORTEC, USA) with the relative efficiency of 35% and resolution of 1.81 keV at 1.33 MeV and ultra-low background shielding with old lead content (ISuS, Sweden) was used for measurement of ^238^U, ^232^Th, and ^40^K. The detector was enclosed in a 10 cm thick compact lead shield. The counting of the samples was obtained by analyzing the spectrum acquired from a multi-channel analyzer (MCA) on a PC with associated software Gamma vision (ORTEC, USA) with 80,000 s counting time.

The full energy absorbed peaks of 351 keV for ^214^Pb and 609 keV for ^214^Bi were identified for calculations of ^238^U activity concentration. The full energy absorbed peaks of 238 keV for ^212^Pb, 581 keV for ^208^Tl, and 911 keV for ^228^Ac were used for ^232^Th, and a single peak of 1460 keV was used for ^40^K. The minimum detectable concentrations (MDCs) of ^238^U, ^232^Th, ^235^U, and ^40^K for this measurement were 5.2 × 10^−3^, 5.2 × 10^−3^, 3.4 × 10^−3^, and 1.5 × 10^−3^ Bq kg^−1^, respectively^47^. We used Eqs. (1) and (2) to calculate activity concentrations from these measurements.

1 $$A=\frac{n}{E Y W{f}_{c}},$$

2 $${L}_{D}={L}_{C}+K{\sigma }_{D},$$

Here *n* is net count rate (cps), *E* is the counting efficiency, *Y* is the photon emission probability, *W* is the sample weight (kg), and *f*_c_ is the correction factor (including summing-in, summing-out, decay factor, recovery factor, attenuation factor, branching ratio, and growth factor), *L*_D_ is the detection limit, *L*_C_ is the decision threshold, ${\sigma }_{D}$ is the standard deviation, and *K* is the error probability^23,48,49^.

### Drinking water quality measurements and identification of heavy metal elements

We collected drinking water samples and measured their temperature, pH and electroconductivity (Laquatwin, Horiba, Japan). Moreover, we measured the ambient dose equivalent rates (PDR-111, Hitachi, Japan) around the collection location. The drinking water samples were acidified to 3% using nitric acid. Furthermore, a triple quadrupole inductively coupled plasma mass spectrometry (ICP-MS-QQQ) instrument (8800, Agilent, USA) was used for determining concentrations of major and heavy metal elements (Cu, Cr, Cd, Mn, Co, As, Se, Ba, ^206^Pb, ^207^Pb, ^208^Pb) and a Dionex ion chromatography system (ICS-210, Thermo Scientific, USA) was used for determining concentrations of ions (F^−^, Cl^−^, NO_3_^−^, Na^+^, and Ca^2+^).

## Radiation exposure assessment

### Indoor-to-outdoor dose-rate ratio

The measurements of indoor and outdoor ambient dose equivalent rates (H^*^(10)) were made using a pocket survey meter (PDR-111, Hitachi, Ltd., Japan). This was done for the high radiation area, and the normal radiation (control) and medium area. GPS was used to locate the measurement points. A total of 450 houses were surveyed: 100 in the control area, 100 in the medium radiation area, and 250 houses in the high radiation area. Around 50 houses were selected from each village in the high radiation area. Indoor and outdoor dose measurements were conducted randomly at the hamlets in a village where people were living at the time of the study. The measurements were taken at the height of 1 m above the ground surface and 2 m from the wall. The pocket survey meter was calibrated annually using a standard source of ^137^Cs with a correction factor (*CF*) of 0.998. The dose-rate can be estimated using Eq. (3).

3 $${H}^{*}(10)={N}_{read} \times CF$$

Here H^*^(10) is ambient dose equivalent rates (nSv h^−1^), *N*_read_ is the value of the ambient dose equivalent rate obtained from the survey meter, and *CF* is the correction factor.

### External annual effective dose estimation

The ambient dose equivalent is an operational quantity for area monitoring. However, for the assessment of the residence dose, an individual dose received by the residents is required, so we conducted individual measurements of the personal dose equivalent *H*_*p*_(10) using optically stimulated luminescence dosimeters (OSLDs, Nagase Landauer, Ltd., Japan). In the high radiation area, 80 dosimeters were used by adults, 20 dosimeters were used in the medium area and 20 dosimeters were used in the control area. The dosimeters were used for a full year, and every user wore it in the daytime, and at nighttime it was placed near the bedside. The detector has been calibrated using the ^137^Cs source with the detector positioned on a phantom.

### Internal radiation dose measurements: ingestion

We collected about 30 food samples of 2 kg each. All samples were foodstuffs commonly consumed daily by residents such as rice, fish, meat, and vegetables. Most of the population in Mamuju consumes mainly locally grown or produced food which comes from traditional markets. Based on these facts, we determined the location and type of food samples commonly and daily consumed after taking into account availability of these foodstuffs. The samples were dried using a freeze dryer (Labconco, USA) at − 70 °C and 10 Pa until dryness (around 3–4 days) and then sieved using a 180 µm mesh filter. The samples were transferred into a standard Marinelli beaker and kept for 30 days to achieve a secular equilibrium before being analyzed using the HPGe detector^50^.

Furthermore, we also measured ^226^Ra and ^222^Rn in drinking water; determination of ^226^Ra was done by a liquid scintillation counting method^21^***, ***a total of 18 drinking water samples of 1 L each and 12 samples from the literature^21^ were collected using PET bottles, filtered using a 0.45 µm pore membrane and adjusted to pH 2 with the addition of 65% nitric acid. After that, a 200 g sample was weighed out and evaporated at around 60 °C until the sample weight was approximately 20 g. From this a sample of approximately 10 mL was transferred into a high-performance glass vial (PerkinElmer, USA) and accurately weighed. After that, a 10 mL aliquot of scintillation cocktail (Ultima Gold uLLT, PerkinElmer, USA) was added and the solution was allowed to stand for 30 days to reach equilibrium with the progeny isotopes.

For ^222^Rn concentration measurement in drinking water, a total of 30 drinking water samples of 250 mL each were collected using a glass bottle and measured with an electrostatic collection type radon monitor (RAD7, Durridge Co., USA) connected to a water analysis accessory (RAD-H2O, Durridge Co., USA). We measured the water samples on days in two seasons, rainy (November 8, 2018) and dry (July 8, 2019). The RAD7 detector was connected to a bubbling kit, for degassing of radon in the water sample into the air in a closed loop. A water sample was put into a radon-tight reagent bottle (250 mL) connected to the closed loop to detect alpha activity. Before the radon gas reached the detector it was dried with desiccant (CaSO_4_, Drierite, W A Hammond, USA) to absorb the moisture. Air was then circulated in the closed loop for a 5 min cycle in 5 recycle times until the radon was uniformly mixed with the air. The measured alpha activity was recorded, from which the radon concentration was obtained directly.

### Internal radiation dose measurements*:* inhalation

We conducted inhalation exposure measurements by measuring radon, thoron and thoron progeny. The measurements of ^222^Rn and ^220^Rn concentrations used the RADUET monitor (Radosys, Ltd, Hungary)^51^. The RADUET had solid-state nuclear track detectors (SSNTDs) made from CR-39 and also a thoron progeny monitor. The radon, thoron and thoron progeny measurements were carried out in the same location together with the measurement of the indoor and outdoor ambient dose-rate. However, those data could not be retrieved from 42 houses because the house owners were no longer living. They were installed in 408 houses: 100 houses in the NBRA, 100 houses in the medium radiation area and 208 houses in the high radiation area. They were placed in the center of the living room of each house or at least 2 m from the wall for a one-year measurement period in which the monitor were replaced at three-month intervals. Every house was well ventilated and had a window in every room. After the exposure, the CR-39 was chemically etched for 24 h in a 6 M NaOH solution at 60 °C, and alpha tracks were counted with an optical microscope^16,52^. The radon and thoron activity concentrations were calculated using the track densities for each of two pieces of CR-39 and the conversion factors for radon and thoron. To measure thoron progeny, we used the stainless steel plate with CR-39 and aluminized film^53,54^.

A RADUET contains paired detection chambers, a low-diffusion chamber, and a high-diffusion chamber. In principle, the track densities in the low-diffusion chamber (*d*_L_) and high-diffusion chamber (*d*_H_) depend on both radon and thoron concentrations in the air. For calculating radon and thoron concentrations, the obtained total track densities are replaced into Eqs. (4) and (5)

4 $${C}_{Rn}=\left({d}_{L}-\overline{b }\right)\times \frac{{f}_{Tn2}}{t.\left({f}_{Rn1}.{f}_{Tn2}-{f}_{Rn2}.{f}_{Tn1}\right)}-({d}_{H}-\overline{b })\frac{{f}_{Tn1}}{t.\left({f}_{Rn1}.{f}_{Tn2}-{f}_{Rn2}.{f}_{Tn1}\right)}$$

5 $${C}_{Tn}=\left({d}_{H}-\overline{b }\right)\times \frac{{f}_{Rn1}}{t.\left({f}_{Rn1}.{f}_{Tn2}-{f}_{Rn2}.{f}_{Tn1}\right)}-({d}_{L}-\overline{b })\frac{{f}_{Rn2}}{t.\left({f}_{Rn1}.{f}_{Tn2}-{f}_{Rn2}.{f}_{Tn1}\right)}$$

6 $$N_{{{\text{TnP}}}} = EETC \times CF_{{{\text{TnP}}}} \times T + b_{{2}}$$

Here *C*_*Rn*_ and *C*_*Tn*_ are the mean concentrations of radon and thoron during the exposure period in Bq m^−3^. *d*_*L*_ and *d*_*H*_ are the total alpha track densities (track m^−2^) taken from the CR-39 detectors of low and high air-exchange rate chambers. *f*_*Rn1*_ and *f*_*Tn1*_ are the radon and thoron calibration coefficients for the low air-exchange rate chamber in tracks m^−2^ kBq^−1^ m^3^ h^−1^. *f*_*Rn2*_ and *f*_*Tn2*_ are the radon and thoron calibration coefficients for the high air-exchange rate chamber in tracks m^−2^ kBq^−1^ m^3^ h^−1^. *t* is the exposure time in hours and $\overline{b }$ is the background track density of the CR-39 detector in tracks m^−2^^52^. The low-diffusion chamber limits the diffusion of thoron into the chamber; therefore, *c*_11_ ≫ *c*_12_. The high-diffusion chamber is designed such that both radon and thoron can diffuse into the chamber easily, and *c*_21_ ≈ *c*_22._ *N*_*TnP*_ is the track density of CR-39 in the thoron progeny deposition detector, EETC is the equilibrium equivalent thoron concentration, and *CF*_TnP_ is the conversion factor for thoron progeny which was 6.9 × 10^–2^ (Bq m^−3^ h)^−1^^55^. Furthermore, for quality control, the radon and thoron monitor must be calibrated. The calibration was carried out in the Institute of Radiation Emergency Medicine, Hirosaki University^56^.

### Estimation of annual effective dose

For quantification of the effective dose, the effective dose was calculated as the accumulation of the dose received from external and internal exposure. We note that the estimated annual effective doses were calculated for adults. External exposure comes from exposure to environmental gamma radiation, while internal exposure can occur through digestion and breathing. Internal exposure through digestion can occur due to the ingestion of food/drink into the body, while internal exposure through breathing can occur due to inhalation of gas containing radioactive substances. An effective dose calculation methodology can use the following equation by UNSCEAR^2^:

9 $${E}_{\mathrm{T}} = {H}_{\mathrm{p}}(d) + \sum_{j}{e}_{j,\mathrm{ing}}{I}_{j,\mathrm{ing}}+ \sum_{j}{e}_{j,\mathrm{inh}}.{I}_{j,\mathrm{inh}}$$

where *E*_T_ is an effective dose (Sv), *H*_p_(d) is the personal dose equivalent due to external exposure obtained by personal dosimeter (Sv) at a depth of 10 mm for penetrating radiation (H_p_(10)), ${e}_{j,\mathrm{ing}}$ is the committed effective dose per unit activity intake by ingestion (Sv Bq^−1^) for radionuclide (*j*) by age group (g), ${e}_{j,\mathrm{inh}}$ the dose coefficient of inhalation dose (Sv Bq^−1^) per unit of respiratory input for radionuclides (*j*) by the age group (g), *I*_*j*,ing_ *I*_*j*,inh_ is the ingestion and inhalation intake (Bq).

10 $${D}_{\mathrm{Rn}}={C}_{\mathrm{Rn}}\times F\times {DCF}_{\mathrm{TnP}}\times OF$$

11 $${D}_{\mathrm{Tn}}=EETC\times {DCF}_{\mathrm{TnP}}\times OF$$

To calculate input through digestion (*I*_*j*,ing_) derived from food, the population consumption data for food types whose radionuclide concentrations are known were based on consumption pattern data in which the rice, meat + vegetable and fruit daily consumptions were 270.4, 64.6 and 36.8 g with one year equal to 365 days^57^. On the other hand, respiratory input (Ij,inh) is the average of calculated radon and thoron concentrations for one-year measurement. To avoid the effect or influence of season, ventillation and so on, the average of radon thoron concentration for one year was used to calculate effective dose with the dose conversion factor for radon of 1.7 × 10^–5^ mSv (Bq h m^−3^)^−1^ and the dose coefficient for thoron of 1.07 × 10^–4^ mSv (Bq h m^−3^)^−1**^^58,59^.

Calculation of external exposure dose assumed that the population received external exposure for 8 h outside the home (from 8 am to 4 pm), considering that almost all residents who lived in the HNBRA are farmers with a total of 7000 h for radon occupancy factor in houses^34,60^. For ingestion, the daily consumption of water was 1.6 L d^−1^ based on the questionnaire survey, and the dose conversion factors for adults are reported as 2.8 × 10^–7^ Sv Bq^−1^ for ^226^Ra, 4.5 × 10^–8^ Sv Bq^−1^ for ^238^U and 2.3 × 10^–7^ Sv Bq^−1^ for ^232^Th^61^.

### Statistical analysis and map creation

We used ORIGIN PRO 2020b (student version, OriginLab Corp, USA) to evaluate significant relationships between the distribution coefficients of radionuclides for characterization exposure. We conducted a Pearson and Spearman rank correlation analysis, and we also calculated the values of correlation coefficients with a two-tailed significance test (*p* value at 0.05). Furthermore, we also compared the results of the dose assessment between the HNBRA and control area using a two-sided student's t test. We used MAPINFO PROFESSIONAL (version 10.5, Precisely, USA) to create maps and dose distribution maps.

### Ethical approval

The study itself was approved by the Committee of Medical Ethics of Hirosaki University Graduate School of Medicine (2018-089, Hirosaki, Japan).

## Supplementary Information

Supplementary Information 1.
