# Supplementary material for: Comprehensive exposure assessments from the viewpoint of health in a unique high natural background radiation area, Mamuju, Indonesia
Source: Sci Rep. 2021 Jul 16;11:14578. doi: 10.1038/s41598-021-93983-2 (PMC8285509; doi:10.1038/s41598-021-93983-2)
Supplement: Supplementary file 1 — Supplementary Information 1. [file 41598_2021_93983_MOESM1_ESM.docx]

**Supplementary Information**

**Title**: Comprehensive Exposure Assessments from The Viewpoint of Health in a Unique High Natural Background Radiation Area, Mamuju, Indonesia

**Authors** : Eka Djatnika Nugraha^1,2,+^, Masahiro Hosoda^2,3,+^, Kusdiana^1^, Untara^1^, June Mellawati^1^, Nurokhim^1^, Yuki Tamakuma^2,3^, Abarrul Ikram^1^, Mukh Syaifudin^1^, Ryohei Yamada^4^, Naofumi Akata^3^, Michiya Sasaki^5^, Masahide Furukawa^6^, Shinji Yoshinaga^7^, Masaru Yamaguchi^2^, Tomisato Miura^3^, Ikuo Kashiwakura^2^, Shinji Tokonami^3,*^

**Corresponding Author**: Shinji Tokonami ([tokonami@hirosaki-u.ac.jp](mailto:tokonami@hirosaki-u.ac.jp))

**Affiliation**:

# ^1^Center for Technology of Radiation Safety and Metrology, National Nuclear Energy Agency, Jl Lebak Bulus Raya No 49, Jakarta Selatan, 12440, DKI Jakarta, Indonesia

# ^2^Department of Radiation Science, Hirosaki University Graduate School of Health Sciences, 66-1 Hon-cho, Hirosaki, 036-8564, Aomori, Japan

# ^3^Institute of Radiation Emergency Medicine, Hirosaki University, 66-1 Hon-cho, Hirosaki, 036-8564, Aomori, Japan

# ^4^Nuclear Fuel Cycle Engineering Laboratories, Japan Atomic Energy Agency, 4-33, Muramatsu, Tokai-mura, Naka-gun, Ibaraki 319-1194,

# ^5^Japan Nuclear Technology Research Laboratory, Central Research Institute of Electric Power Industry, 2-11-1, Iwadokita, Komae-shi, Tokyo 201-8511 Japan

# ^6^Graduate School of Engineering and Science, University of the Ryukyus, 1 Senbaru, Nishihara-cho, Okinawa 903-0213, Japan

# ^7^Research Institute for Radiation Biology and Medicine, Hiroshima University, 1-2-3, Kasumi, Minami-ku, Hiroshima 734-8553, Japan


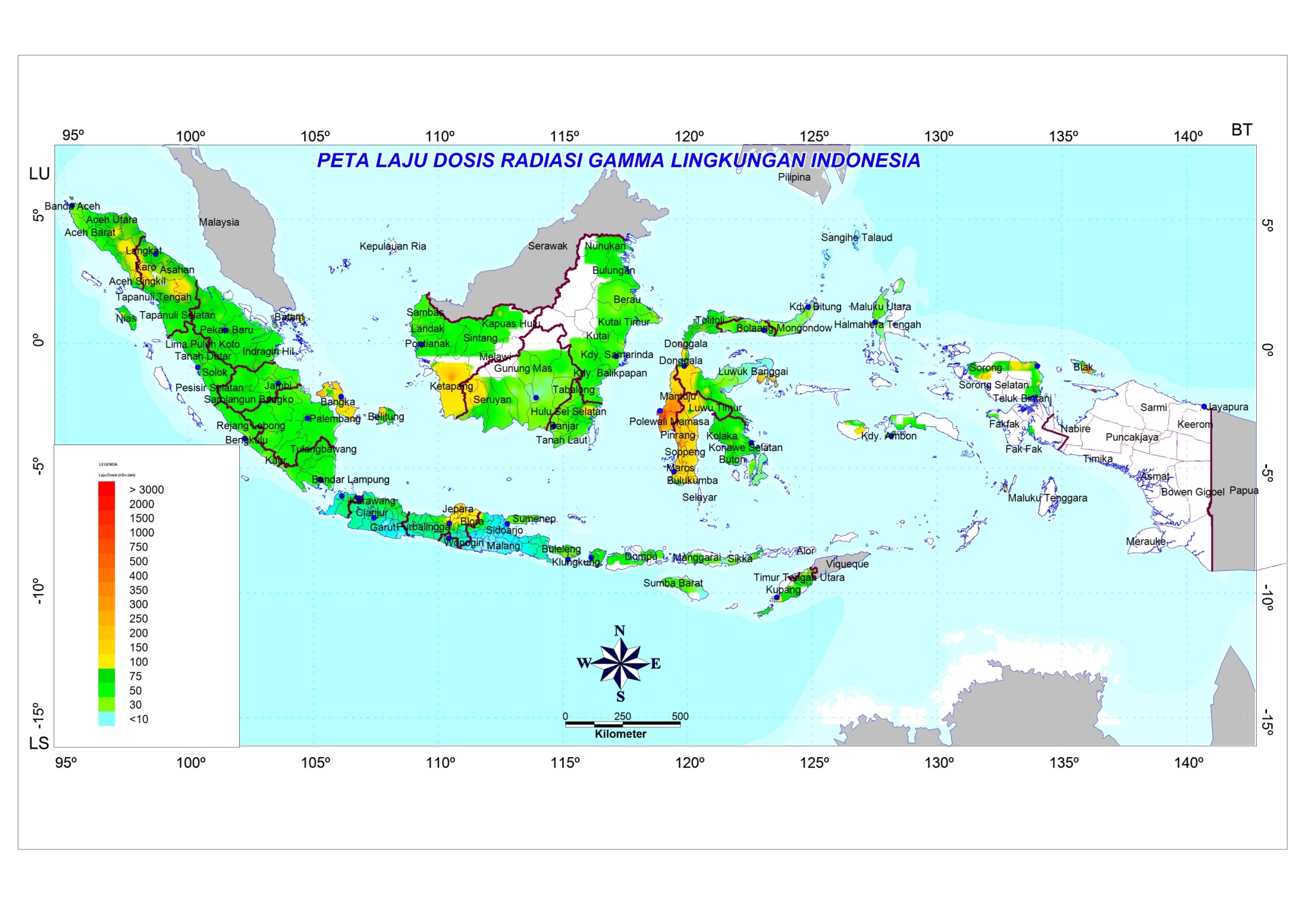


**Dose rate ( nSv Gy^-1^)**

**AMBIENT GAMMA RADIATION DOSE RATE MAP**

**Figure S1**. Ambient gamma radiation map of Indonesia ^14^. Obtained from the National Nuclear Energy Agency of Indonesia, Center for Technology of Radiation Safety and Metrology. (http://sadarlingkungan.batan.go.id/berkas/kti/makalah//M_PTKMR_RE_0_KTINasionalDalamBentukBuku_PTKMR-BATAN_PetaPaparanRadiasiGammaIndonesia_210529192004.pdf)

**Figure. S2** Average activity concentration of natural radioactivity in soil samples from study villages.

**Figure. S3** Source identification; (a) correlation analysis between ambient dose rate and ^238^U, (b) correlation analysis between ambient dose rate and ^232^Th, (c) correlation analysis between ambient dose rate and ^40^K.

**Table S1.** Concentrations of natural radionuclides in soil samples collected in the Mamuju study area

| Village | Location | Ambient dose rate  (nGy h^-1^) | Radionuclide concentration (Bq kg^-1^) | | | |
| --- | --- | --- | --- | --- | --- | --- |
|  |  |  | ^238^U | ^232^Th | ^40^K | ^235^U |
| Northern Botteng | A1 | (1.33 ± 0.10) ×10^3^ | (2.92 ± 0.28) ×10^3^ | (3.54 ± 0.28) ×10^3^ | (2.00 ± 0.20) ×10^2^ | (2.10 ± 0.30) ×10^1^ |
|  | A2 | (1.38 ± 0.11) ×10^3^ | (2.89 ± 0.27) ×10^3^ | (3.58 ± 0.28) ×10^3^ | (1.98 ± 0.20) ×10^2^ | (2.01 ± 0.20) ×10^1^ |
|  | A3 | (1.03 ± 0.08) ×10^3^ | (6.34 ± 0.60) ×10^2^ | (1.15 ± 0.11) ×10^3^ | (4.55 ± 0.44) ×10^2^ | (5.01 ± 0.50) ×10^0^ |
|  | A4 | (1.02 ± 0.08) ×10^3^ | (6.30 ± 0.59) ×10^2^ | (1.13 ± 0.11) ×10^3^ | (4.38 ± 0.42) ×10^2^ | (4.02 ± 0.50) ×10^0^ |
|  | A5 | (1.32 ± 0.10) ×10^3^ | (1.37 ± 0.13) ×10^3^ | (2.05 ± 0.19) ×10^3^ | (2.98 ± 0.29) ×10^2^ | (1.01 ± 0.10) ×10^1^ |
|  | A6 | (1.24 ± 0.99) ×10^3^ | (1.36 ± 0.13) ×10^3^ | (1.97 ± 0.19) ×10^3^ | (3.16 ± 0.30) ×10^2^ | (1.00 ± 0.10) ×10^1^ |
|  | A7 | (1,32 ± 0.10) ×10^3^ | (1.78 ± 0.17) ×10^3^ | (3.15 ± 0.28) ×10^3^ | (2.27 ± 0.22) ×10^2^ | (1.30 ± 0.15) ×10^1^ |
|  | GM | (1.23 ± 0.98) ×10^3^ | (1.41 ± 0.14) ×10^3^ | (2.15 ± 0.21) ×10^3^ | (2.89 ± 0.27) ×10^2^ | (1.02 ± 0.10) ×10^1^ |
| Takandeang | B1 | (1.98 ± 0.16) ×10^3^ | (2.00 ± 1.90) ×10^3^ | (3.31 ± 0.25) ×10^3^ | (2.76 ± 0.27) ×10^2^ | (1.30 ± 0.2) ×10^1^ |
|  | B2 | (1.77 ± 0.14) ×10^3^ | (1.98 ± 0.15) ×10^3^ | (3,23 ± 0.25) ×10^3^ | (2.62 ± 0.25) ×10^2^ | (1.30 ± 0.2) ×10^1^ |
|  | B3 | (7.36 ± 0.59) ×10^2^ | (5.70 ± 0.54) ×10^2^ | (9.75 ± 0.30) ×10^2^ | (3.00 ± 0.25) ×10^2^ | (4.00 ± 0.5) ×10^0^ |
|  | B4 | (9.85 ± 0.79) ×10^2^ | (6.16 ± 0.50) ×10^2^ | (1.25 ± 0.11) ×10^3^ | (2.81 ± 0.21) ×10^2^ | (4.00 ± 0.5) ×10^0^ |
|  | GM | (1.26 ± 0.10) ×10^3^ | (1.09 ± 0.11) ×10^3^ | (1,90 ± 0.19) ×10^3^ | (2.79 ± 0.22) ×10^2^ | (7.00 ± 0.9) ×10^0^ |
| Botteng | C1 | (7.00 ± 0.56) ×10^2^ | (1.32 ± 0.15) ×10^3^ | (1.12 ± 0.11) ×10^3^ | (3.80 ± 0.37) ×10^2^ | (9.00 ± 0.11) ×10^1^ |
|  | C2 | (8.30 ± 0.66) ×10^2^ | (3.42 ± 0.23) ×10^3^ | (9.22 ± 0.87) ×10^2^ | (1.89 ± 0.19) ×10^2^ | (1.70 ± 0.21) ×10^2^ |
|  | C3 | (8.90 ± 0.71) ×10^2^ | (3.45 ± 0.23) ×10^3^ | (9.11 ± 0.88) ×10^2^ | (1.90 ± 0.18) ×10^2^ | (1.80 ± 0.22) ×10^2^ |
|  | C4 | (6.30 ± 0.50) ×10^2^ | (1.02 ± 0.96) ×10^3^ | (1.12 ± 0.11) ×10^3^ | (1.47 ± 0.16) ×10^2^ | (7.00 ± 0.81) ×10^2^ |
|  | C5 | (5.70 ± 0.46) ×10^2^ | (1.22 ± 0.14) ×10^3^ | (1.12 ± 0.11) ×10^3^ | (1.21 ± 0.13) ×10^2^ | (9.00 ± 1.00) ×10^2^ |
|  | C6 | (5.80 ± 0.46) ×10^2^ | (1.26 ± 0.13) ×10^3^ | (1.12 ± 0.13) ×10^3^ | (2.06 ± 0.20) ×10^2^ | (9.01 ± 1.00) ×10^2^ |
|  | C7 | (5.40 ± 0.43) ×10^2^ | (1.09 ± 0.13) ×10^3^ | (1.11 ± 0.12) ×10^3^ | (2.33 ± 0.24) ×10^2^ | (8.00 ± 0.80) ×10^2^ |
|  | GM | (6.66 ± 0.43) ×10^2^ | (1.60 ± 0.15) ×10^3^ | (1.06 ± 0.11) ×10^3^ | (1.97 ± 0.18) ×10^2^ | (1.01 ± 0.10) ×10^2^ |
| Taan | D1 | (7.10 ± 0.57) ×10^2^ | (1.09 ± 0.11) ×10^3^ | (8.19 ± 0.71) ×10^2^ | (5.55 ± 0.55) ×10^2^ | (8.00 ± 0.80) ×10^0^ |
|  | D2 | (7.00 ± 0.56) ×10^2^ | (1.19 ± 0.11) ×10^3^ | (8.89 ± 0.77) ×10^2^ | (5.51 ± 0.51) ×10^2^ | (8.00 ± 0.80) ×10^0^ |
|  | D3 | (6.90 ± 0.58) ×10^2^ | (9.98 ± 0.92) ×10^2^ | (8.56 ± 0.78) ×10^2^ | (5.22 ± 0.51) ×10^2^ | (7.03 ± 0.80) ×10^0^ |
|  | GM | (7.00 ± 0.56) ×10^2^ | (1.09 ± 0.11) ×10^3^ | (8.54 ± 0.78) ×10^2^ | (5.42 ± 0.53) ×10^2^ | (8.01 ± 0.80) ×10^0^ |
| Ahu | E1 | (7.30 ± 0.58) ×10^2^ | (7.68 ± 0.76) ×10^2^ | (8.65 ± 0.88) ×10^2^ | (2.02 ± 0.20) ×10^2^ | (6.00 ± 0.80) ×10^0^ |
|  | E2 | (6.30 ± 0.50) ×10^2^ | (7.77 ± 0.71) ×10^2^ | (8.66 ± 0.88) ×10^2^ | (2.02 ± 0.20) ×10^2^ | (6.00 ± 0.80) ×10^0^ |
|  | E3 | (6.20 ± 0.50) ×10^2^ | (6.89 ± 0.66) ×10^2^ | (8.87 ± 0.88) ×10^2^ | (2.02 ± 0.20) ×10^2^ | (5.00 ± 0.50) ×10^0^ |
|  | GM | (6.58 ± 0.53) ×10^2^ | (7.44 ± 0.73) ×10^2^ | (8.73 ± 0.86) ×10^2^ | (2.02 ± 0.20) ×10^2^ | (6.00 ± 0.50) ×10^0^ |
| Salugatta | F1 | (1.05 ± 0.80) ×10^2^ | (6.40 ± 0.60) ×10^1^ | (8.90 ± 0.90) ×10^1^ | (6.77 ± 0.64) ×10^2^ | (2.00 ± 0.20) ×10^0^ |
|  | F2 | (9.90 ± 0.80) ×10^1^ | (5.50 ± 0.40) ×10^1^ | (8.50 ± 0.80) ×10^1^ | (6.55 ± 0.64) ×10^2^ | (2.00 ± 0.20) ×10^0^ |
|  | F3 | (1.15 ± 0.90) ×10^2^ | (6.40 ± 0.50) ×10^1^ | (9.90 ± 0.90) ×10^1^ | (6.66 ± 0.62) ×10^2^ | (2.00 ± 0.20) ×10^0^ |
|  | GM | (1.06 ± 0.80) ×10^2^ | (6.10 ± 0.50) ×10^1^ | (9.10 ± 0.80) ×10^1^ | (6.66 ± 0.63) ×10^2^ | (2.00 ± 0.20) ×10^0^ |
| Topoyo (NBRA control area) | X1 | (6.50 ± 0.50) ×10^1^ | (3.80 ± 0.30) ×10^1^ | (5.80 ± 0.50) ×10^1^ | (5.55 ± 0.51) ×10^2^ | (2.00 ± 0.20) ×10^0^ |
|  | X2 | (5.10 ± 0.40) ×10^1^ | (4.30 ± 0.40) ×10^1^ | (5.60 ± 0.60) ×10^1^ | (5.51 ± 0.53) ×10^2^ | (2.00 ± 0.20) ×10^0^ |
|  | X3 | (7.00 ± 0.60) ×10^1^ | (4.10 ± 0.30) ×10^1^ | (6.00 ± 0.60) ×10^1^ | (5.43 ± 0.52) ×10^2^ | (2.00 ± 0.20) ×10^0^ |
|  | GM | (6.10 ± 0.50) ×10^1^ | (4.10 ± 0.30) ×10^1^ | (5.80 ± 0.50) ×10^1^ | (5.50 ± 0.51) ×10^2^ | (2.00 ± 0.20) ×10^0^ |

**Table S2**. Radioactivity in foodstuff samples

| Food group | Food sample/ Collection location | Radionuclide concentration (Bq kg^-1^) | | |  |
| --- | --- | --- | --- | --- | --- |
|  |  | ^226^Ra | ^232^Th | ^40^K | |
| Rice Group | Rice/Northern Botteng | <MDC | <MDC | (2.54 ± 0.31) ×10^1^ | |
|  | Rice/Botteng | <MDC | (6.20 ± 1.2) ×10^0^ | (2.82 ± 0.29) ×10^2^ | |
|  | Rice/Ahu | (0.30 ± 0.01) ×10^0^ | <MDC | (3.20 ± 0.40) ×10^1^ | |
|  | Rice/Traditional market | (0.40 ± 0.01) ×10^0^ | <MDC | (1.20 ± 0.10) ×10^1^ | |
| Average | | (0.40 ± 0.01) ×10^0^ | (6.20 ± 1.20) ×10^0^ | (8.79 ± 0.93) ×10^1^ | |
| Meat/  Vegetables/  Fish Group | Spinach/Northern Botteng | (3.81 ± 1.30) ×10^0^ | (1.81 ± 0.12) ×10^0^ | (1.52 ± 0.15) ×10^3^ | |
|  | Luffa/Northern Botteng | (1.28 ± 0.25) ×10^1^ | (1.22 ± 0.28) ×10^1^ | (1.36 ± 0.13) ×10^3^ | |
|  | Cassava/Popanga | (5.01 ± 0.90) ×10^0^ | (1.19 ± 0.21) ×10^1^ | (3.10 ± 0.30) ×10^2^ | |
|  | Chicken/Botteng | (1.05 ± 0.30) ×10^0^ | (0.21 ± 0.03) ×10^0^ | (3.52 ± 0.35) ×10^2^ | |
|  | Red snapper/Fish market | (1.22 ± 0.30) ×10^0^ | (0.42 ± 0.06) ×10^0^ | (1.60 ± 0.16) ×10^2^ | |
|  | Spinach/Botteng | (5.3 ± 1.50) ×10^0^ | (0.63 ± 0.19) ×10^0^ | (8.77 ± 0.88) ×10^2^ | |
|  | Cassava/Botteng | (1.02 ± 0.15) ×10^1^ | (2.01 ± 0.13) ×10^1^ | (4.26 ± 0.42) ×10^2^ | |
|  | Spinach/Botteng | (1.01 ± 0.15) ×10^2^ | (5.05 ± 0.11) ×10^1^ | (6.06 ± 0.52) ×10^3^ | |
|  | Cassava leaves/Botteng | (1.20 ± 0.25) ×10^1^ | (6.20 ± 0.13) ×10^1^ | (7.35 ± 0.90) ×10^2^ | |
|  | Chicken/Botteng | <MDC | (0.51 ± 0.10) ×10^0^ | (3.52 ± 0.32) ×10^1^ | |
|  | Tempeh (fermented soybean)/Traditional market | (6.01 ± 1.00) ×10^0^ | (1.35 ± 0.20) ×10^1^ | (7.03 ± 0.80) ×10^1^ | |
|  | Spinach/Takandeang | (5.84 ± 0.72) ×10^1^ | (6.72 ± 0.82) ×10^1^ | (6.45 ± 0.66) ×10^2^ | |
|  | Cassava/Takandeang | (2.70 ± 0.26) ×10^1^ | (1.19 ± 0.11) ×10^2^ | (4.95 ± 0.47) ×10^2^ | |
|  | Cassava leaves/Botteng | (6.00 ± 0.61) ×10^1^ | (2.55 ± 0.24) ×10^2^ | (6.70 ± 0.64) ×10^2^ | |
|  | Tilapia/Fish market | (1.10 ± 0.17) ×10^1^ | (1.40 ± 0.14) ×10^1^ | (2.02 ± 0.18) ×10^3^ | |
|  | Chicken Takandeang | (1.50 ± 0.15) ×10^2^ | (2.81 ± 0.27) ×10^2^ | (9.74 ± 0.91) ×10^2^ | |
|  | Long bean leaves/Botteng | (4.50 ± 0.45) ×10^1^ | (1.05 ± 0.11) ×10^2^ | (1.10 ± 0.11) ×10^3^ | |
|  | Cassava/Taan | (2.01 ± 0.20) ×10^0^ | (2.01 ± 0.20) ×10^0^ | (1.57 ± 0.15) ×10^2^ | |
|  | Spinach/Ahu | (3.80 ± 0.30) ×10^1^ | (7.01 ± 0.70) ×10^1^ | (1.05 ± 0.10) ×10^3^ | |
|  | Cassava leaves/Taan | (1.35 ± 0.12) ×10^2^ | (5.6 ± 0.60) ×10^1^ | (4.07 ± 0.38) ×10^2^ | |
| Average | | (3.60 ± 0.65) ×10^1^ | (5.71 ± 0.66) ×10^1^ | (9.71 ± 0.91) ×10^2^ | |
| Fruits Group | Papaya/Northern Botteng | (3.03 ± 0.50) ×10^0^ | (4.41 ± 0.80) ×10^0^ | (7.67 ± 0.82) ×10^1^ | |
|  | Banana/Northern Botteng | (1.01 ± 0.30) ×10^0^ | (0.29 ± 0.10) ×10^0^ | (6.45 ± 0.62) ×10^2^ | |
|  | Banana/Botteng | (2.42 ± 0.29) ×10^1^ | (2.10 ± 0.80) ×10^0^ | (4.70 ± 0.46) ×10^2^ | |
|  | Banana/Takandeang | (5.01 ± 0.50) ×10^0^ | (2.01 ± 2.00) ×10^1^ | (3.25 ± 0.31) ×10^2^ | |
|  | Cacao/Takandeang | (7.10 ± 1.00) ×10^1^ | (1.25 ± 1.70) ×10^1^ | (5.57 ± 0.53) ×10^2^ | |
|  | Cacao/Ahu | (4.02 ± 0.20) ×10^1^ | (5.02 ± 0.30) ×10^0^ | (2.70 ± 0.25) ×10^2^ | |
| Average | | (7.41 ± 0.90) ×10^0^ | (7.41 ± 0.97) ×10^0^ | (3.90 ± 0.37) ×10^2^ | |
